# Salt Consumption in Latvian Population: A Pilot Study

**DOI:** 10.3390/medicina54010010

**Published:** 2018-03-25

**Authors:** Ilva Lazda, Māris Goldmanis, Inese Siksna

**Affiliations:** 1 Department of Risk Assessment and Epidemiology, Institute of Food Safety, Animal Health and Environment BIOR, Lejupes Street 3, LV-1076 Riga, Latvia; inese.siksna@bior.lv; 2 Department of Economics, Royal Holloway University of London, Egham TW20 0EX, UK; maris.goldmanis@gmail.com

**Keywords:** sodium, potassium, 24-h urine, salt intake, dietary data, urinary data

## Abstract

*Background and objective*: High dietary sodium intake is associated with multiple health risks, and the average sodium intake in Latvia is higher than the World Health Organization has recommended. In Latvia, no study so far has combined self-reported dietary data on sodium and potassium intake with objective measurements in 24-h urine samples. This pilot study aimed to cross-validate both methods and to assess any possible factors interfering with the collection of samples and data in large, population-based future studies of sodium and potassium intake in Latvian adults. *Materials and methods*: A stratified random sample of healthy Latvian adults aged 19–64 (*n* = 30) was drawn. Dietary data of sodium and potassium was collected using one 24-h dietary recall and a two-day food diary. Sodium and potassium excretion was measured by one 24-h urinary collection. *Results*: Median intake of sodium and potassium based on dietary data was 2276.4 mg/day (interquartile range (IQR), 1683.3–3979.4) and 2172.0 mg/day (IQR, 1740.6–3506.5), respectively. Median intake of sodium and potassium based on urinary data was 3500.3 mg/day (IQR, 2191.0–5535.0) and 2965.4 mg/day (IQR, 2530.2–3749.9), respectively. Urinary data showed significantly higher results than dietary records (Wilcoxon signed rank test, *p* = 0.023). Only 13% of the subjects did not exceed the WHO-recommended limit of 2000 mg of sodium per day, and only 33% consumed at least the recommended allowance of 3510 mg of potassium per day. Median intake of salt was 8.8 g/day (IQR, 5.5–13.8) (according to urinary data). *Conclusions*: The findings from the present study showed considerable underestimation of dietary sodium and potassium intake based on self-reported dietary data. Urinary data revealed more accurate results, and showed that Latvian adults exceed the amount of salt recommended and consume less potassium than recommended. The pilot study also showed that the chosen methods are adequate for implementation in large, population-based studies to evaluate dietary intake of salt, sodium, and potassium in populations of Latvian adults.

## 1. Introduction

Morbidity due to noncommunicable diseases (NCDs), such as cardiovascular diseases, cancers, chronic respiratory diseases and diabetes, is increasing each year worldwide. NCDs are also one of the leading causes of death in the world. Previous studies have demonstrated that increased intake of salt can increase blood pressure, which is a significant risk factor of cardiovascular disease [1].

People in Europe are currently consuming more than the World Health Organization’s (WHO) recommended 5 g of salt or 2 g of sodium per day [2]. In most European countries, national averages of daily salt intake range from 8 to 12 g [1]. However, lower salt consumption is usually reported in countries where data is collected using dietary surveys [3]. Food consumption data in Latvia was first obtained in 2009. The results showed that the mean consumption of salt was 7.1 g per day [4], suggesting that Latvia could be ranked among those European countries that consume relatively less salt. Food consumption data cannot be considered completely accurate, because information regarding the amount of added salt in industrial products may not reflect the actual salt content in the product [1]. In accordance with the applicable regulations [5] and guidelines [6], the packaging of food products in Latvia must contain information on salt content; however, this can vary noticeably and may reach as low as 40% of the actual salt content. Food consumption data may also suffer from inaccurately evaluated amounts of salt added by people in the preparation process of food at home due to imprecise units of measurement (pinch, teaspoon, etc.) and recall errors [1].

Meanwhile self-reported food data still remains important in evaluating food sources of salt. In addition to food data, 24-h urinary collection analysis should be made. Based on sodium excretion through kidneys, it is considered the “gold standard” in the monitoring of consumed salt [7,8]. In addition to sodium, it is also important to evaluate the amount of excreted potassium, which can be associated with the consumption of fruits and vegetables. By increasing the amount of potassium in the diet, it is possible to reduce blood pressure and the risk of developing cardiovascular disease, stroke, and coronary heart disease [9].

There are no previous studies on salt consumption based on 24-h urinary collection analysis in Latvia; therefore, the main aims of the present pilot study were to validate the chosen methods, to identify any difficulties in their implementation, and to detect initial patterns in the data. We also aim to apply the methodology developed during the present pilot study to future studies on a much larger nationally representative sample.

## 2. Materials and Methods

### 2.1. Study Design and Participants

The present cross-sectional pilot study was conducted in 2015 in Latvia. It included 30 participants aged 19–64, living in different regions of Latvia. Participants were recruited through the general practices at which they were registered using multistage stratified cluster sampling. In the first stage of sampling, a representative sample of Latvia’s general practices was established and stratified in six strata based on the location of the practice. In the second stage, individual participants were randomly selected from the patient register of every general practice and stratified in six strata based on age and sex [10].

This study was conducted according to the guidelines laid down in the Declaration of Helsinki and Convention for the protection of Human Rights and Dignity of the Human Being with regard to the Application of Biology and Medicine: Convention on Human Rights and Biomedicine; all procedures were approved by the Ethical Commission of the Institute of Food Safety, Animal Health and Environment “BIOR”. Written informed consent was obtained from all subjects and caregivers. The privacy rights of the subjects were observed.

The exclusion factors were as follows: unsigned consent form for participation in the study, chronic liver disease, kidney or heart failure, stroke, women during pregnancy, and patients taking diuretics over the past two months.

### 2.2. Dietary Record

During the first meeting with the interviewer, the procedure of the study was explained, after which a series of questionnaires were completed, including (1) a food frequency questionnaire; (2) a 24-h dietary recall about the day before the meeting; and (3) a questionnaire on the subject’s socio-demographic background, health status, and eating habits. The latter included questions about salt usage habits and the frequencies of cooking at home and eating out. At the end of the meeting, a two-day food diary was given to the participants to fill in on the 24-h urine collection day and the day before it. Food diaries were gathered during the second meeting with the interviewer together with a 24-h urinary sample.

To make the comparison with urinary data adequate, dietary data for only one day from the two-day food diary (for the day when 24-h urine was collected) was used in calculating the dietary data on sodium and potassium intake [11]. Dietary data was compared with the WHO recommendations of consuming no more than 2000 mg of sodium or 5 g of salt per day [2], and consuming at least 3510 mg of potassium per day [9].

### 2.3. 24-h Urinary Collection

During the first meeting with the interviewer, participants were asked to complete a 24-h urine collection. Urinary collection and storage containers were provided. The participants were instructed to sample the first morning void separately and then to collect all urine over the following 24 h, including the first void on the next morning. The time of the beginning and end of the collection was recorded in a questionnaire, along with notes on any discarded urine and medicines used. Women were asked not to collect the urine during their menstruation.

During the second meeting, both urine sample containers—the 24-h urine sample and spot urine sample—were handed over to the interviewer. Collections were considered valid if the volume of the 24-h sample was at least 500 mL [12]. Samples were analyzed in three different laboratories using two different methods to compare the accuracy and reliability of different methods and to evaluate the most cost-effective method, which could be used in the future for larger studies. Sodium and potassium were analyzed using ion-selective electrode method and inductively coupled plasma mass spectrometry. To evaluate best the method and laboratory for testing samples, some samples were sent twice to laboratories under different ID numbers to detect measurement stability. 20% of samples were analyzed twice.

To compare the amount of sodium and potassium in the urine with food consumption data, analytical values of excreted sodium and potassium mass were multiplied by coefficients of 1/0.9 and 1/0.7 respectively, assuming that 90% of ingested sodium and 70% of ingested potassium is excreted in the urine [12]. Urinary data was compared with the WHO recommendations of consuming no more than 2000 mg of sodium or 5 g of salt per day [2], and consuming at least 3510 mg of potassium per day [9].

### 2.4. Anthropometric Measures

Anthropometric measures (body weight, height, hip circumference, waist circumference and waist-to-hip ratio) were made by the interviewer or the general practitioner. Two consecutive standard blood pressure measurements for each hand were made by the general practitioner, ensuring a 5 min rest in a seated position prior the measurements to avoid ‘white coat effect’ bias. The average values of the readings were used in further analysis [13].

### 2.5. Statistical Analysis

All statistical analyses were performed using IBM SPSS version 22.0 (IBM, Armonk, NY, USA); figures were generated using Stata version 14.0 (StataCorp LLC, College Station, TX, USA). A *p*-value below α = 0.05 was considered to indicate statistical significance. Descriptive statistics were used to describe frequencies, mean values, standard deviations, median values and interquartile dispersions. The Mann-Whitney U test was performed for differences between independent samples, the Wilcoxon signed-rank test was used for paired samples, the Kruskal-Wallis test was carried out for multiple independent samples, and the Pearson χ^2^ test was applied to test for independence of categorical variables.

## 3. Results

The study sample comprised fifteen subjects of each sex. Demographic characteristics of the study sample are presented in Table 1. The mean age of the subjects was 43.6, with a standard deviation of 12.1, and most of them (63.4%) were overweight. More women (66.7%) than men (26.7%) were with tertiary education.

One 24-h recall and a two-day food diary were collected from all subjects. All dietary data was used to determine the main sources of sodium and potassium in the subjects’ diet, but dietary data for only one day from the two-day food diary (for the day that the 24-h urine was collected) was used in calculating the dietary data on sodium and potassium intake (Table 2). According to the dietary data, 43% of the subjects did not consume more than 2000 mg of sodium per day. At the same time, only 23.3% of the subjects consumed at least 3510 mg of potassium per day.

All of the 24-h urine collections were considered valid (the volume of the sample was at least 500 mL). The results (Table 3) revealed that significantly fewer subjects—13%—did not exceed the intake of 2000 mg of sodium per day than the dietary data showed. Meanwhile, the consumption of potassium was revealed to be higher according to urinary data—33% of the subjects consumed at least 3510 mg of potassium per day.

**Figure 1:**
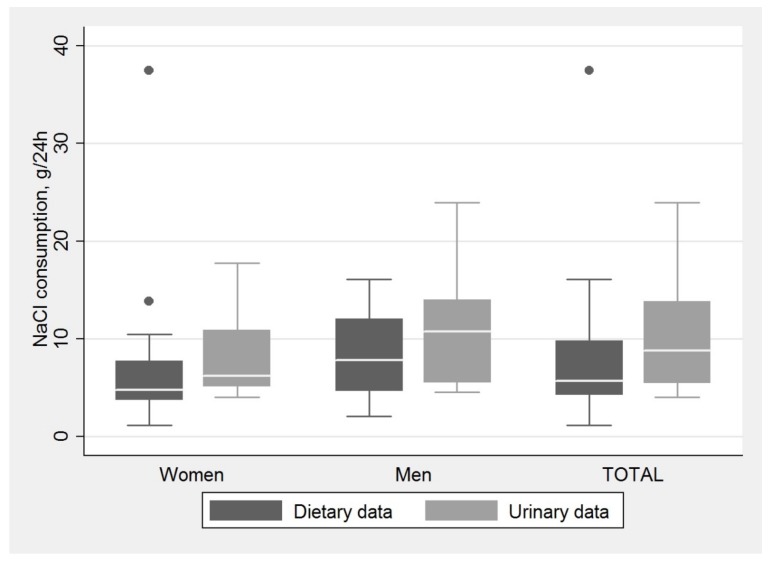
Median salt intake (g/24 h) for women (*n* = 15), men (*n* = 15) and total count of subjects (*n* = 30) according to urinary and dietary data. (The box indicates the interquartile range; horizontal line in the box, median; error bars, maximum and minimum values, excluding outliers; dots (circles), outliers).

The obtained urinary and dietary data on sodium was converted to consumption of salt. Median values of salt consumption according to both urinary and dietary data are presented in Figure 1. The median values of salt consumed exceeded 5 g of salt per day for all groups, with only one exception—dietary data of salt consumption in women. In general, urinary data showed higher intake of salt than dietary data (Wilcoxon signed-ranks test, *p* = 0.023); the median value of salt consumption according to urinary data in all subjects was 8.8 (interquartile range (IQR), 5.5–13.8) g of salt per day, compared to 5.7 (IQR, 4.2–9.9) g of salt according to dietary data. Differences were not significant at the chosen significance level of α = 0.05 when comparing urinary and dietary data for each sex separately—for men (Wilcoxon signed-rank test, *p* = 0.053) and for women (Wilcoxon signed-rank test, *p* = 0.112). This observation could be due to the small sample size of the pilot study.

Sodium and potassium samples were analyzed in three different laboratories using two different methods—the ion-selective electrode method and inductively coupled plasma mass spectrometry (Table 4 and Table 5).

Differences in levels were not statistically significant according to the Wilcoxon matched-pairs signed-ranks test (*p* = 0.491). Differences in variances (standard deviations) were not statistically significant according to Levene’s robust test (*p* = 0.913).

The main sources of salt in the subjects’ diet, according to one 24-h recall and a two-day food diary, were added salt (including spice mixes containing salt) (31% of total sodium consumed), processed meat products (16%), fried and pickled fish (8%), dark (6%) and white (6%) bread and cheese (5%).

The principal sources of potassium were vegetables (except potatoes; 18% of total potassium consumed), fruits and berries (14%), potatoes (10%), meat (8%), milk and milk products (8%), processed meat products (7%), coffee (6%) and deserts, sweets and pastry (5%).

The results suggest that women consume less salt than men; however, the difference was not significant at the chosen significance level of α = 0.05 (Mann-Whitney *U* test, *p* = 0.071), likely due to the small sample size of the pilot study. Eating habits of women and men showed that all of the women cooked their food at home at least several times a week, while 20% of men never cooked at home. Spice mixes with salt were most preferred spices for 40% of women and 27% of men, and there were no women or men who would not use salt at all when cooking at home.

It was also observed that subjects with tertiary education were less likely to exceed the recommended levels of salt consumption than were those without a college degree (Mann-Whitney *U* test, *p* = 0.02); 21% of subjects with tertiary education and only 6% of subjects with less than tertiary education consumed no more than 2000 mg of sodium a day.

The obtained data on the amount of sodium consumed did not show any significant connection with the frequency of meals outside home (Kruskal-Wallis test, *p* = 0.801), or with the frequency of cooking at home (Kruskal-Wallis test, *p* = 0.447). The same was observed for potassium (Kruskal-Wallis test, *p* = 0.524, *p* = 0.944, respectively). 

**Figure 2:**
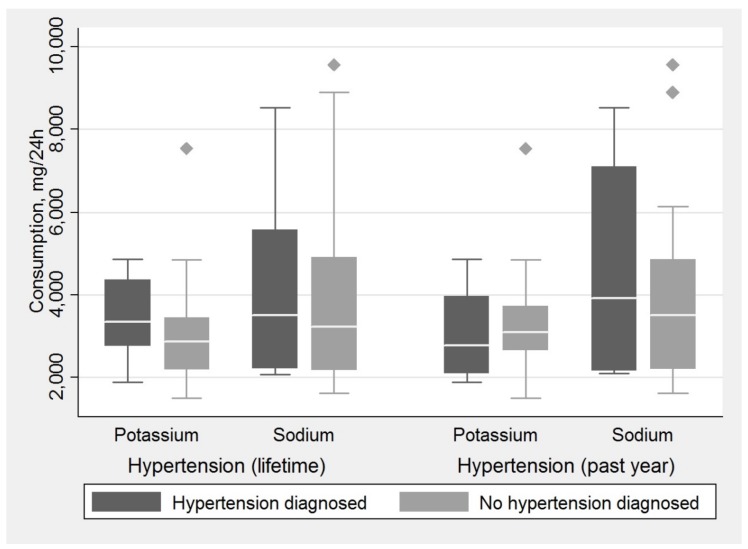
Median intake of sodium and potassium (mg/24 h) according to urinary data for subjects with diagnosed hypertension (*n* = 12) and subjects with no diagnosis of hypertension (*n* = 18). (The box indicates the interquartile range; horizontal line in the box, median; error bars, maximum and minimum values, excluding outliers; dots (cubic circles), outliers).

Data regarding medical history of hypertension of the subjects was compared with the urinary data of sodium and potassium intake (Figure 2). We observed a pattern of subjects with a history of hypertension during their lifetime consuming more sodium and potassium than those with no such history, albeit with no statistical significance (Mann-Whitney *U* test, *p* = 0.703 for sodium; *p* = 0.253 for potassium). Subjects with hypertension diagnosed in the past 12 months consumed more sodium but less potassium than those with no diagnosis of hypertension in the past 12 months, with no statistical significance (Mann-Whitney *U* test, *p* = 0.641 in both cases). It is worth noting that all groups consumed excessive amounts of sodium and inadequate amounts of potassium.

The link between sodium consumption and hypertension appeared stronger if an indicator variable of exceeding the WHO-recommended threshold was used instead of the exact level of sodium consumption. In our sample, none of the subjects with adequate sodium intake (≤2000 mg/24 h) had ever been diagnosed with hypertension, while 46% of the subjects with increased sodium intake (≥2000 mg/24 h) had had such a diagnosis. The effect was not significant at the chosen significance level of α = 0.05 (Pearson χ^2^ test, *p* = 0.079).

## 4. Discussion

There are no previous studies on sodium and potassium consumption based on 24-h urine collection in Latvia. Previously obtained food consumption data in Latvia from 2009 showed that the average consumption of salt, based on dietary data, was 7.1 g per day [4]. Today, when 24-h urine collection is considered the “gold standard” in monitoring consumed salt [7], we can assess the amount of consumed salt more accurately. Our pilot study revealed that Latvians consume 8.8 g of salt per day, which exceeds the World Health Organization’s recommended 5 g per day [2]. Comparing our results to those representing other European adults, Latvian adults tend to consume less salt (8.8 g/day) compared to Spanish adults (9.8 g/day) [14] and Belgian adults in different regions of the country (10.7 g/day and 9.9 g/day) [15], but more salt than English adults (8.1 g/day) [16].

All of the studies described, as well as ours, show that men consume more salt and sodium than women. This could be explained by men consuming more food or women being more health-conscious than men. However, data on eating habits revealed that eating less outside home does not ensure healthier habits towards salt consumption. Since the tests we performed (the Kruskal-Wallis test) have low power in small samples, our failure to detect any links between eating out and the intake of sodium and potassium should not be taken as conclusive evidence of the absence of such links in the Latvian population. Further studies in larger samples are necessary to investigate the relationship between eating habits and sodium and potassium intake [17,18].

Meanwhile, self-reported food data shows significantly lower median salt consumption—5.7 g of salt per day, which exceeds the WHO’s recommended daily amount [2] by only 14%, while the obtained amount from the urinary data exceeds it by 76%. This observation can be linked with difficulties in estimating the amount of salt added in the process of preparing food at home due to imprecise units of measurement (pinch, teaspoon, etc.) and recall errors, and also in processed foods, since it can vary noticeably, reaching as low as 40% of the actual salt content [5,6]. The underestimated amount of salt intake by self-reported dietary data is well described [19,20].

Since 24-h urine collection is considered a more accurate method of estimating the amount of salt (and also potassium) consumed, we use urinary data as our primary estimates of consumption. Nonetheless, dietary data still remains important in evaluating food sources of salt. Our results show that not only should the amount of salt added in the process of cooking be reduced, but increased attention should be paid to the consumption of processed foods like processed meat and fish products, bread and cheese. This data corresponds to the WHO’s assessment that processed foods tend to be the main contributors to salt intake [1].

In our study, urinary data also reveals higher amount of potassium consumed, but in this case it is a positive turn—33.3% (instead of 23.3% from dietary data) of the subjects consumed an adequate amount of potassium, which is above 3510 mg per day, according to the WHO [9]. Nevertheless, the men in our study consumed a median amount of 3420.8 mg of potassium per day and the women 2787.0 mg, which correspond to the lowest rates from the European Prospective Investigation into Cancer and Nutrition (EPIC) study [21]. That study investigated potassium intake in ten European countries, and the lowest intake of potassium in both men and women was found in Greece (3536 mg/day and 2730 mg/day). The highest rates were found in different regions of Spain—4870 mg/day for men and 3723 mg/day for women. Such a disparity with our data could be linked with lower availability of main sources of potassium, such as fresh fruit and vegetables, in the Latvian diet throughout the year due to a colder climate.

The EPIC study showed that meat and meat products, cereals and cereal products, non-alcoholic beverages and vegetables were the main sources of potassium for men and nonalcoholic beverages, vegetables, fruits, dairy foods and products, and cereals and cereal products for women. Although fresh fruit, berries and vegetables were the main sources of potassium in our study, taking the low urinary rates of potassium in account, it can be assumed that consumption of fresh fruit and vegetables is still too low and should be promoted alongside salt reduction plans.

In our study, a tentative association was observed between a high consumption of sodium (≥2000 mg/day) and lifetime prevalence of hypertension. This association did not quite reach the conventional threshold of statistical significance, presumably due to the small sample size. The suggested effects of sodium and potassium consumption on hypertension in the Latvian population merits further investigation in larger samples, which would not suffer from the limited statistical power of the present pilot study. Although we did not observe a high significance in our study sample, other studies show that the prevalence of hypertension in the European population is 30–45% [22] and suggest the implementation of salt-reduction plans. Currently, there is no national salt-reduction strategy in Latvia, but since it is a member state of the WHO, Latvia provides the WHO with information on the current salt policy [1], organizes informative campaigns at the national level, and has implemented regulations in allowed amount of salt in foods served in educational institutions, social care and social rehabilitation institutions [23]. In order to achieve more successful public health benefits, a new strategy should emphasize reducing the amount of sodium added to foods and promote consumption of potassium rich foods and reducing the amount of processed and packaged foods, instead of restricting sodium alone [24].

To analyze sodium and potassium samples, two different methods—ion-selective electrode method and inductively coupled plasma mass spectrometry—were used in our pilot study. Given the small sample sizes and the limited number of repeated measurements, no definite conclusions can be drawn about differences between measurements obtained using ion-selective and mass spectrometry methods.

Although for all calculations 24-h urine samples alone were used, spot urine samples were also analyzed for the same elements. Correlation between 24-h urinary sodium and spot urinary sodium were checked. In line with findings of validation studies by other authors in a variety of populations (Brazil, China, Korea), we found that in the Latvian population, the formulas of Tanaka [25] and Kawasaki [26] produce estimates of 24-h urinary excretion of sodium that are highly correlated with the measured values, but do not show good agreement on an individual level. In particular, we observed that the biases of the estimates strongly decrease with the level of sodium excretion. In fact, the formulas produce overestimates for low levels of sodium excretion and underestimates for high levels of sodium excretion. We conclude that formulas for estimating 24-h urinary excretion of sodium from measurements in urine spot samples need to be recalibrated for use in the Latvian population.

The main contribution of our study is the use of 24-h urine collection in combination with dietary data. This is, to the best of our knowledge, the first such attempt in any study of the Latvian population. Recent studies have suggested that even 24-h urine collections have little utility in estimating daily salt intake in any given individual and that sodium is excreted in an infradian fashion even when salt intake is rigidly fixed [27]. Further analysis of the same data showed that seven 24-h collections would be necessary to separate salt intake of 6 g/day from 9 g/day or from 12 g/day [28]. Similar variabilities were observed for potassium [29]. It has also been advised that multiple 24-h collections over time would be necessary when conducting clinical trials of salt reduction [30]. Nevertheless, 24-h urine collection remains to be the “gold-standard” in estimating population salt intake [7].

A potential limitation of our study is our sample size [11], but this is an inherent problem in any pilot study. The main aims of the present study were to validate and fine-tune the chosen methods, to identify any difficulties in their implementation, and to detect initial patterns in the data. We deem these goals to have been achieved successfully. We will apply the methodology developed during the present study to future studies on much larger nationally representative samples, which will hopefully yield more conclusive insights into the patterns tentatively identified in this pilot study.

## 5. Conclusions

The findings from the present study showed considerable underestimation of dietary sodium and potassium intake based on self-reported dietary data. Urinary data revealed more accurate results and showed that Latvian adults as a mean population have higher sodium intake and lower potassium intake than the WHO has recommended.

The pilot study showed that the chosen methods were adequate for implementation in a large, population-based study to evaluate dietary intake of salt, sodium, and potassium in the Latvian adult population.

## Figures and Tables

**Table 1:** Demographic characteristics of the study sample.

Characteristic	Men (*n* = 15)	Women (*n* = 15)	Total (*n* = 30)
Age, mean (SD), years	44.5 (11.8)	42.8 (12.8)	43.6 (12.1)
Age, %			
19–35 years	26.7	33.3	30.0
36–50 years	33.3	33.3	33.3
51–64 years	40.0	33.3	36.7
Height, mean (SD), cm	181.9 (3.9)	165.8 (6.9)	173.9 (9.9)
Weight, mean (SD), kg	95.0 (18.3)	74.5 (16.4)	84.7 (20.0)
BMI, mean (SD), kg/m^2^	28.7 (5.8)	27.2 (5.9)	27.9 (5.8)
BMI category, %			
Underweight (16–18.49 kg/m^2^)	0	6.7	3.35
Normal weight (18.5–24.9 kg/m^2^)	33.3	33.3	33.3
Overweight (25–45 kg/m^2^)	66.7	60	63.4
Waist-hip ratio, mean (SD)	0.96 (0.08)	0.83 (0.12)	0.89 (0.12)
Education, %			
Tertiary	26.7	66.7	46.7
Other	73.3	33.3	53.3
Ever-diagnosed hypertension, %			
Yes	46.7	33.3	40.0
No	53.3	66.7	60.0
Hypertension diagnosed in past year, %			
Yes	26.7	13.3	20.0
No	73.3	86.7	80.0

SD—standard deviation; BMI—body mass index.

**Table 2:** Dietary data on sodium and potassium intake by sex.

Analyzed criterion	Men (*n* = 15)	Women (*n* = 15)	Total (*n* = 30)
Sodium, medium (IQR), mg/day	3142.9 (1875.5–4814.0)	1900.1 (1507.9–3080.8)	2276.4 (1683.3–3979.4)
% of subjects consuming no more than 2000 mg/day *	33.3	53.3	43.3
Potassium, medium (IQR), mg/day	2151.2 (1779.6–3586.5)	2421.4 (1489.1–3323.7)	2172.0 (1740.6–3506.5)
% of subjects consuming at least 3510 mg/day **	26.7	20.0	23.3

IQR—interquartile range. * According to the WHO guidelines for sodium intake [2]; ** According to the WHO guidelines for potassium intake [9].

**Table 3:** Urinary data on sodium and potassium consumption by sex.

Analyzed criterion	Men (*n* = 15)	Women (*n* = 15)	Total (*n* = 30)
Sodium, medium (IQR), mg/day	4299.8 (2245.1–5579.2)	2487.6 (2074.3–4345.1)	3500.3 (2191.0–5535.0)
% of subjects consuming no more than 2000 mg/day *	6.7	20.0	13.3
Potassium, medium (IQR), mg/day	3420.8 (2932.0–3960.9)	2787.0 (2131.9–3598.0)	2965.4 (2530.2–3749.9)
% of subjects consuming at least 3510 mg/day **	40.0	26.7	33.3

IQR—interquartile range. * According to the WHO guidelines for sodium intake [2]; ** According to the WHO guidelines for potassium intake [9].

**Table 4:** Measurements obtained for sodium in 24-h samples.

Analyzed Criterion	Ion-Selective Electrode Method (*n* = 30)	Inductively Coupled Plasma Mass Spectrometry (*n* = 30)
Sodium level, mean (SD), mmol/L	103.6 (55.3)	102.2 (58.2)

SD—standard deviation.

**Table 5:** Measurements obtained for potassium in 24-h samples.

Analyzed criterion	Ion-Selective Electrode Method (*n* = 30)	Inductively Coupled Plasma Mass Spectrometry (*n* = 30)
Potassium level, mean (SD), mmol/L	40.7 (18.4)	39.8 (15.7)

Differences in levels were not statistically significant according to the Wilcoxon matched-pairs signed-ranks test (*p* = 0.165). Differences in variances (standard deviations) were not statistically significant according to Levene’s robust test (*p* = 0.516). SD—standard deviation.
